# Efficient Gene Knockout and Knockdown Systems in Neospora caninum Enable Rapid Discovery and Functional Assessment of Novel Proteins

**DOI:** 10.1128/msphere.00896-21

**Published:** 2022-01-12

**Authors:** Tiago W. P. Mineo, Jessica H. Chern, Amara C. Thind, Caroline M. Mota, Santhosh M. Nadipuram, Juan A. Torres, Peter J. Bradley

**Affiliations:** a Department of Microbiology, Immunology and Molecular Genetics, University of California, Los Angeles, Los Angeles, California, USA; b Laboratory of Immunoparasitology “Dr. Mário Endsfeldz Camargo,” Institute of Biomedical Sciences, Universidade Federal de Uberlândia, Uberlândia, Minas Gerais, Brazil; c Molecular Biology Institute, University of California, Los Angeles, Los Angeles, California, USA; University at Buffalo

**Keywords:** *Neospora caninum*, *Toxoplasma gondii*, Ku80, CRISPR/Cas9, dense granule, auxin-inducible degron

## Abstract

The development of molecular genetics has greatly enhanced the study of the biology and pathology associated with parasites of the phylum Apicomplexa. While the molecular tools are highly developed for the apicomplexan Toxoplasma gondii, the closely related parasite Neospora caninum lacks efficient tools for genetic manipulation. To enable efficient homologous recombination in N. caninum, we targeted the Ku heterodimer DNA repair mechanism in the genomic reference strain, Nc-Liverpool (NcLiv), and show that deletion of *Ku80* results in a destabilization and loss of its partner Ku70. Disruption of *Ku80* generated parasites in which genes are efficiently epitope tagged and only short homology regions are required for gene knockouts. We used this improved strain to target novel nonessential genes encoding dense granule proteins that are unique to N. caninum or conserved in T. gondii. To expand the utility of this strain for essential genes, we developed the auxin-inducible degron system for N. caninum using parasite-specific promoters. As a proof of concept, we knocked down a novel nuclear factor in both N. caninum and T. gondii and showed that it is essential for survival of both parasites. Together, these efficient knockout and knockdown technologies will enable the field to unravel specific gene functions in N. caninum, which is likely to aid in the identification of targets responsible for the phenotypic differences observed between these two closely related apicomplexan parasites.

**IMPORTANCE** Neospora caninum is a parasite with veterinary relevance, inducing severe disease in dogs and reproductive disorders in ruminants, especially cattle, leading to major losses. The close phylogenetic relationship to Toxoplasma gondii and the lack of pathogenicity in humans drives an interest of the scientific community toward using N. caninum as a model to study the pathogenicity of T. gondii. To enable this comparison, it is important to develop efficient molecular tools for N. caninum, to gain accuracy and save time in genetic manipulation protocols. Here, we have developed base strains and protocols using the genomic reference strain of N. caninum to enable efficient knockout and knockdown assays in this model. We demonstrate that these tools are effective in targeting known and previously unexplored genes. Thus, these tools will greatly improve the study of this protozoan, as well as enhance its ability to serve as a model to understand other apicomplexan parasites.

## INTRODUCTION

Neospora caninum is a coccidian that belongs to a diverse group of parasitic protozoans of the phylum Apicomplexa. N. caninum is closely related to Toxoplasma gondii, which is perhaps the best studied member of the phylum and an important human pathogen of immunocompromised patients and congenitally infected neonates. While T. gondii infects a wide range of warm-blooded vertebrates, including an estimated one-third of the world’s human population (1), N. caninum has a narrower host range, primarily targeting ruminants and dogs (2). It is one of the leading causes of bovine abortions in the world, inducing significant economic losses to cattle raising (3).

One strategy routinely used to gain a better understanding of the biology of these parasites is based on the development of a robust set of molecular approaches that enable reliable analyses of specific genetic targets. For functional analyses, reverse genetics methods were made possible by the advent of transfection protocols in a number of model apicomplexans (4, 5). This advance led to the development of an array of genetic tools for detailed genetic manipulation of these parasites. These tools are best developed and easiest to use in T. gondii, which includes an assortment of promoters, selectable markers, fluorescent proteins, genome editing strategies, and knockout and conditional knockdown approaches (6).

For N. caninum, however, this toolkit lags substantially behind its more prominent, closely-related parasite, T. gondii. Although transfection of *Neospora* has been available for decades, most genetic approaches took advantage of vectors developed specifically for T. gondii (7, 8). While those tools are useful for the stable insertion of heterologous genes of interest (GOI) (9–11), further development of the model is needed. For example, while a background strain with a single point mutation on the hypoxanthine-xanthine-guanine phosphoribosyl transferase gene (Nc1Δ*hxgprt*) has been developed as a selectable marker (12–14), it was generated by chemical mutagenesis, which likely generated many mutations and is only available in the NC-1 strain, not the sequenced reference strain NcLiv.

*HXGPRT* has been used successfully as a selectable marker for stable transformation in T. gondii for over 20 years, due to its safety and versatility conferred by the positive or negative selection with mycophenolic acid/xanthine (Mpa/X) or 6-thioxanthine (6TX), respectively (15). Other available selectable markers include *chloramphenicol acetyltransferase* (*CAT*) and a mutated version of *dihydrofolate reductase-thymidylate synthase* (*DHFR*) driven by either *Toxoplasma* or endogenous *Neospora* promoters. These markers have been effectively used for gene insertions and endogenous tagging, based on selection strategies using resistance to chloramphenicol and pyrimethamine, respectively (16, 17).

The advent of genome editing tools based on clustered regularly interspaced short palindromic repeats-associated gene 9 (CRISPR/Cas9) system has dramatically changed each system in which it has been developed. The CRISPR/Cas9 system was adapted for T. gondii (18, 19) and has contributed to the field by promoting fast and precise targeted gene disruption, endogenous epitope tagging, and site-specific insertion of selectable markers. Without further adaptation, plasmids with CRISPR/Cas9 components used in T. gondii were satisfactorily used to disrupt GFP in N. caninum tachyzoites stably expressing the fluorescent marker, as well as disrupt the *NcGRA7* gene by insertion of a selectable marker (20). Since then, pU6 plasmids—adapted or not with N. caninum promoters—have been employed for endogenous epitope tagging and gene disruption, especially of genes encoding dense granule proteins (21–25). Those strategies typically rely on large homology flanks (over 800 bp) for homology-directed repair or disruption by insertion of selectable markers, which is a setback compared with the stage of development of the CRISPR/Cas9 system found in the *Toxoplasma* model.

The key difference in *Toxoplasma* is the combination of an efficient CRISPR/Cas9 system in the background of a knockout of the *Ku80* gene, which enables highly efficient recombination (26, 27). The Ku heterodimer is conserved in eukaryotes and is formed by two subunits, Ku70 and Ku80, that maintain genomic integrity through binding to DNA double-strand breaks, making repairs by the non-homologous end joining (NHEJ) pathway (28). In its absence, random integration is greatly diminished, which allows for a more precise manipulation of the targeted loci through homologous recombination. In this work, we aimed to further improve the molecular toolbox of the *Neospora* model system. To do this, we disrupted *Ku80* in the NcLiv strain and subsequently disrupted the *HXGPRT* selectable marker used for *Ku80* disruption, thereby recycling this marker for downstream selections. We examined the effect of the absence of Ku80 on its partner Ku70 and demonstrated that CRISPR/Cas9 based endogenous gene tagging and knockouts become efficient similar to that observed in T. gondii. We additionally modified this strain for conditional knockouts by adapting the auxin-inducible degron system. We demonstrate the utility of these strains by disrupting two secreted GRA proteins and conditionally disrupting a novel nuclear protein. Thus, these new tools dramatically improve the molecular toolkit of N. caninum.

## RESULTS

### Disruption of *HXGPRT* in the NcLiv strain using CRISPR/Cas9.

**FIG 1:**
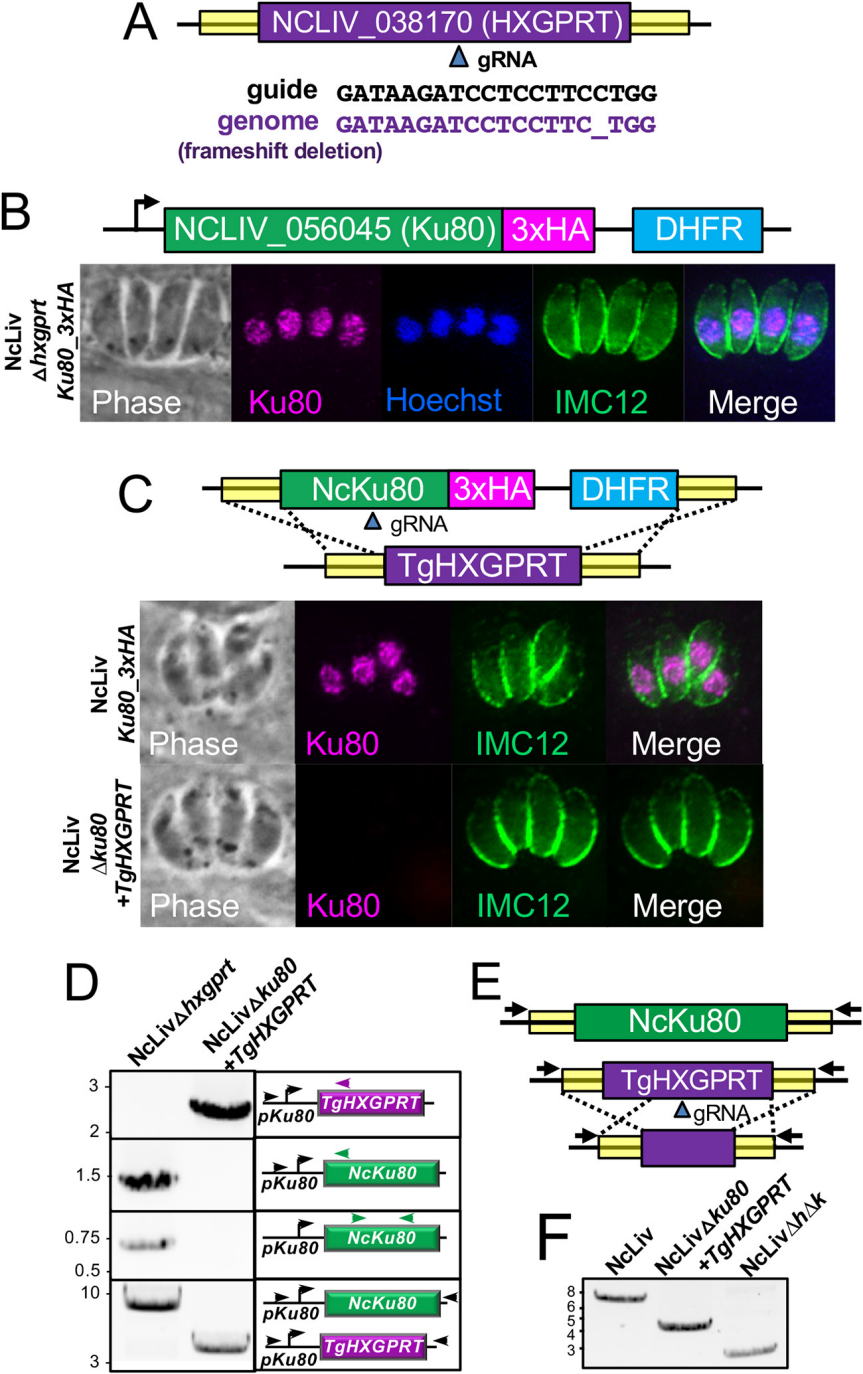
Disruption of *HXGPRT* and *Ku80* in NcLiv strain parasites. (A) Diagram showing CRISPR/Cas9 targeting of *HXGPRT* and resulting deletion of a single nucleotide rendering parasites sensitive to Mpa/X. (B) Schematic and IFA of 3xHA endogenous gene tagging of Ku80. Ku80 localizes to the nucleus as assessed by Hoechst costain, and IMC12 is used to mark the periphery of the parasites. (C) Disruption of tagged *Ku80* using CRISPR/Cas9 and a homology template with the selectable marker *HXGPRT*. IFA shows the lack of staining in the NcLivΔ*ku80* *(+HXGPRT)* strain. (D) PCR verification of the *Ku80* knockout shows the selectable marker is in the *Ku80* locus (primers P47/P48) and the coding region is eliminated in the knockout (primers P47/P49; P50/P51). (E) Diagram and (F) PCR verification (primers P47/P52) showing the elimination of *HXGPRT* using negative selection with 6TX to generate parasites lacking both *Ku80* and *HXGPRT* (denoted NcLiv*ΔhΔk*).

To aid in the genetic manipulation of N. caninum, we first sought to disrupt the selectable marker *HXGPRT* using CRISPR/Cas9 (Fig. 1A). To do this, we generated sequences encoding a guide RNA (gRNA) against *Neospora*’s *HXGPRT* (*NcHXGPRT*, NCLIV_038170) in the pU6 universal plasmid and transfected it into wild-type NcLiv parasites (18, 19). Knockouts of *NcHXGPRT* were negatively selected with 6TX and cloned by limiting dilution, and a clone was chosen that failed to grow in positive selection (Mpa/X). Sequencing of the coding region showed a single nucleotide deletion at the gRNA site within the *HXGPRT* locus (Fig. 1A), and this strain was designated NcLivΔ*hxgprt*.

### Epitope tagging and disruption of Ku80 in N. caninum.

To improve the efficiency of homologous recombination in the NcLivΔ*hxgprt* strain, we then targeted the *Ku80* gene (NCLIV_056045), which has been shown to dramatically improve recombination in T. gondii (26, 27). We first used CRISPR/Cas9 to add a 3xHA epitope tag at the C-terminus of Ku80 as well as the selectable marker *DHFR*, which resulted in parasites with nuclear staining as expected (NcLivKu80^3xHA^; Fig. 1B).

To disrupt the *Ku80* gene in NcLivKu80^3xHA^ background, we created a gRNA against the beta-barrel domain of Ku80 and a homology directed repair template that consisted of the *Ku80* 5′ and 3′ flanking regions, cloned upstream and downstream of a *Toxoplasma HXGPRT* selection cassette (Fig. 1C). These were transfected into the tagged parasites and selected, and clonal isolates were screened for the absence of Ku80 staining. One isolate was selected and the correct replacement of *Ku80* with *HXGPRT* was verified by PCR (Fig. 1D). To improve the utility of this NcLivΔ*ku80* (*+TgHXGPRT*) strain for downstream analyses, we used a similar strategy of a gRNA and homologous recombination to delete the T. gondii *HXGPRT* selectable marker from the *Ku80* locus using negative selection (6TX; Fig. 1E). The deletion of *TgHXGPRT* was confirmed by PCR and the strain lacking both *Ku80* and *HXGPRT* was designated NcLivΔ*hΔk* (Fig. 1F).

### Ku70 is destabilized in NcLiv*ΔhΔk* parasites.

**FIG 2:**
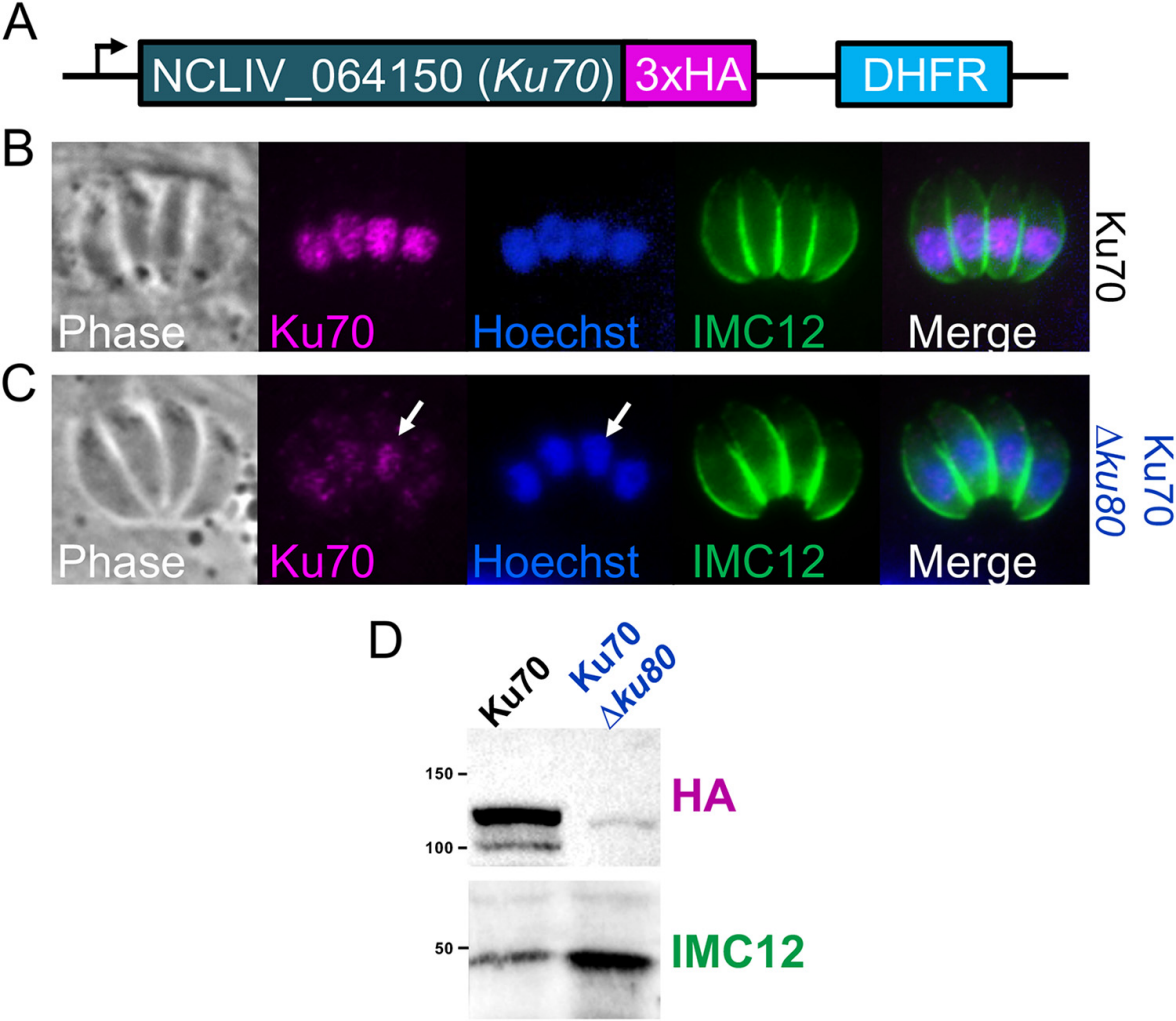
Disruption of Ku80 destabilizes its partner Ku70. (A) Diagram and IFA of Ku70 tagged with and without Ku80. Ku70 is nearly undetectable in the Δ*ku80* parasites. Parasites were costained with Hoechst to stain the nucleus and anti-IMC12 to label the periphery of the parasites. (B) Western blot showing that Ku70 is dramatically reduced in parasites lacking Ku80. IMC12 is used as a loading control.

Ku80 works in conjunction with its partner protein Ku70 to facilitate nonhomologous end joining (28). To assess whether disruption of Ku80 had an effect on its partner, we epitope tagged Ku70 in both NcLivΔ*hxgprt* and NcLivΔ*hΔk* strain parasites (Fig. 2A). Immunofluorescence assay (IFA) of the NcLivΔ*hxgprt* strain containing wild-type Ku80 showed a nuclear localization for Ku70, as expected (Fig. 2B). However, in NcLivΔ*hΔk* strain parasites, the staining of Ku70 was dramatically decreased (Fig. 2C) and in many parasites was undetectable (not shown). The impact on Ku70 was confirmed by Western blot analysis, which showed a nearly complete loss of detectable signal (Fig. 2D). These data indicate that disruption of *Ku80* results in the concomitant destabilization and degradation of Ku70.

### Highly efficient gene tagging in Δ*ku80* parasites identifies novel secreted and nuclear factors.

**FIG 3:**
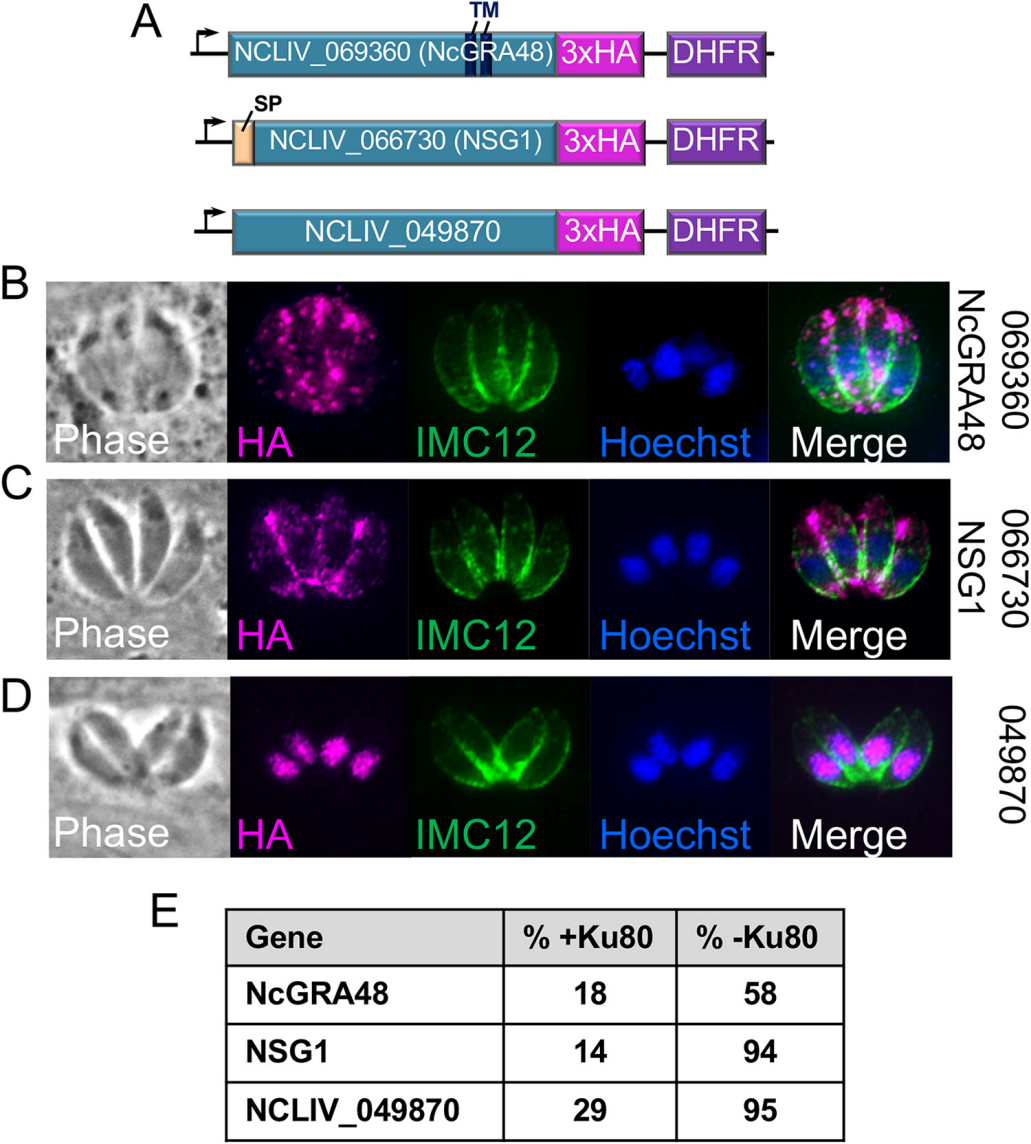
Efficient CRISPR/Cas9 mediated gene tagging in NcLiv*ΔhΔk* parasites identifies new secreted and nuclear proteins. (A) Gene model diagram of endogenous tagging of NCLIV_069360/NcGRA48, NCLIV_066730/NSG1, and NCLIV_049870. GRA48 contains two internal predicted transmembrane domains (TM), and NCLIV_066730 contains a predicted signal peptide (SP). Models are not drawn to scale. (B) IFA showing that NcGRA48 localizes to the vacuolar space surrounding the parasites, as expected for a dense granule protein. IMC12 is used to label the periphery of the parasites, and Hoechst staining is used to detect the parasite’s nucleus. (C) IFA showing that 3xHA tagged NCLIV_066730 localizes to the vacuole. As NCLIV_066730 is *Neospora*-specific, it was named *Neospora*-specific GRA protein 1 (NSG1). (D) IFA showing that NCLIV_049870 localizes to the parasite’s nucleus as shown by colocalization with Hoechst staining. (E) Diagram showing the frequency of endogenous gene tagging with and without *Ku80*. The frequency of gene tagging is dramatically increased in parasites lacking *Ku80*.

Disruption of *Ku80* in T. gondii results in a dramatic increase in the efficiency of CRISPR/Cas9 epitope tagging due to increased homologous recombination (26). To determine if this was also the case in N. caninum, we compared the efficiency of CRISPR/Cas9-mediated epitope tagging of endogenous genes in strains with and without the protein (Fig. 3). We selected three genes for epitope tagging; the *Neospora* orthologue of the T. gondii secreted protein GRA48 (NcGRA48, NCLIV_069360), a novel *Neospora*-specific protein that contains a signal peptide and a downstream series of tandem repeats (NCLIV_066730; Fig. S1 in the supplemental material), and a novel protein (NCLIV_049870) whose orthologue in T. gondii has a strongly negative fitness score in the genome-wide CRISPR/Cas9 screen (TGGT1_235420, -4.8), suggesting it is either important for fitness or essential (Fig. 3A). For epitope tagging, the NcLivΔ*hxgprt* and NcLivΔ*hΔk* strains were transfected with a gRNA construct for each gene as well as a homology repair template with 40 bp regions of homology to the GOI as is frequently used in T. gondii (29). As expected, NcGRA48 tagging showed that this protein localizes to the parasitophorous vacuole (Fig. 3B). Similarly, NCLIV_066730 also localized to the vacuole, indicating that this is indeed a novel *Neospora*-specific GRA protein (Fig. 3C). To our knowledge, NCLIV_066730 is the first *Neospora*-specific GRA protein that has been identified, and thus we named it *Neospora*-specific GRA protein 1 (NSG1) to differentiate it from the other GRA proteins with orthologues in the family. In contrast, NCLIV_049870 localized to the nucleus of the parasite (Fig. 3D). In each case, the efficiency of tagging was dramatically increased from around 20% with Ku80 present to as much as 95% tagging efficiency in Δ*ku80* parasites (Fig. 3E). These data indicate that the NcLivΔ*hΔk* strain has significantly improved recombination, verifies dense granule localization for NcGRA48, and identifies two completely new proteins in N. caninum.

### Disruption of *Ku80* results in efficient gene knockouts with minimal flanking regions.

**FIG 4:**
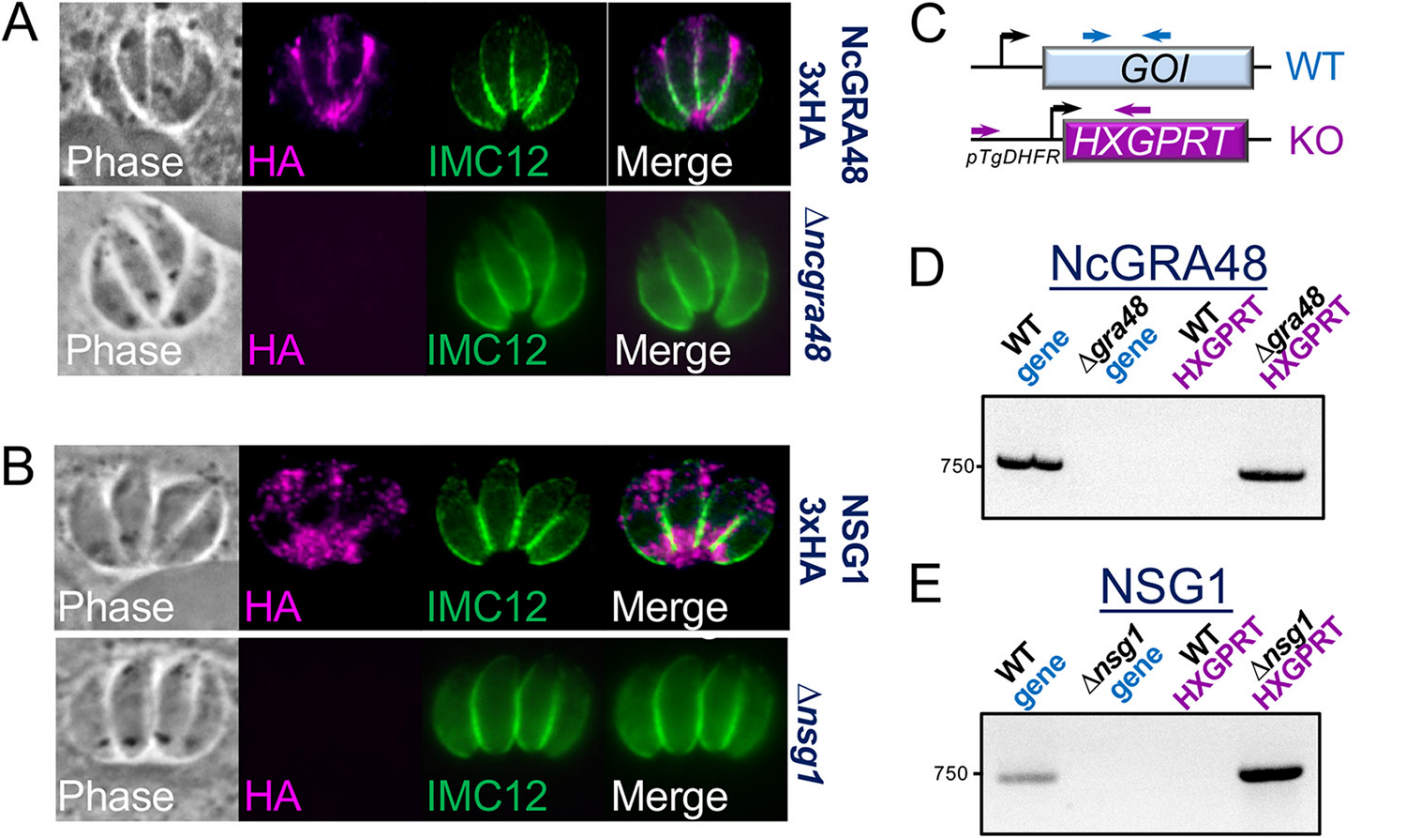
Deletion of *Ku80* enables efficient gene knockouts with minimal flanking regions for homology-directed repair. (A, B) IFA showing that deletion strategies eliminated the 3xHA-tag from NCLIV_069360/NcGRA48 and NCLIV_066730/NSG1 tagged strains. IMC12 is used to stain the periphery of the parasites. (C) Diagram showing strategy to PCR verify the gene of interest (GOI) deletions in NcLiv*ΔhΔk* parasites. (D, E) Agarose gel analyses of the gene knockouts showing integration of *HXGPRT* into the correct locus (NcGRA48: primers P53/P48; NSG1: primers P56/P48) as well as the absence of the GOI coding region in the knockout parasites (NcGRA48: primers P54/P55; NSG1: primers P57/P58).

In T. gondii, disruption of *Ku80* enables highly efficient CRISPR/Cas9-mediated whole gene deletions using a CRISPR/Cas9 gRNA targeting the GOI plus a homology-directed repair template consisting of 40 bp flanking homology regions and a selectable marker (29, 30). In the absence of a *Ku80* deficient line in *Neospora*, gene knockouts have been shown to require substantial flanking regions of homology (21–23). Our knockout attempts using 40 bp homology flanks in a *Ku80* positive line appeared to generate disrupted genes by the insertion of the cassette containing the selectable marker inside the GOI’s coding region, rather than the desired full gene deletions (not shown). To determine if precise deletions of genes could be obtained in NcLivΔ*hΔk* parasites, we attempted to disrupt *NcGRA48* and *NSG1* in the epitope tagged strains using the identical approach with a *Toxoplasma HXGPRT* cassette as the selectable marker (Fig. 4). As predicted, we were able to efficiently disrupt both genes, as shown by a lack of staining for the epitope tag (Fig. 4A and B). We then PCR verified that the entire gene was properly deleted and replaced by *HXGPRT* (Fig. 4C to E). These data demonstrate that whole gene deletions can easily be obtained in NcLivΔ*h*Δ*k* parasites using minimal flanking regions, similar to that seen in T. gondii (29).

### Development of the auxin-inducible degron system for N. caninum.

**FIG 5:**
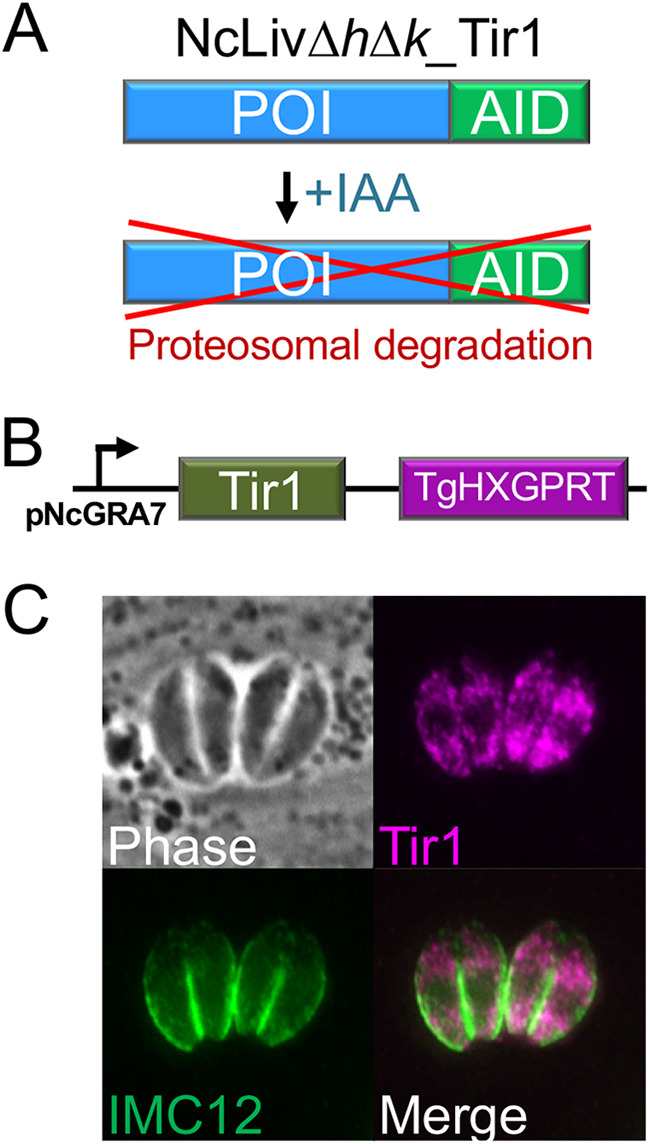
Stable expression of Tir1 in NcLiv*ΔhΔk* parasites. (A) Diagram of the AID system showing an auxin-inducible degron translational fusion is produced by endogenous gene tagging. Addition of indoleacetic acid (IAA) results in proteosomal degradation of the fusion protein. (B) Diagram of Tir1 driven from the *Neospora* GRA7 promoter, with *Toxoplasma HXGPRT* as a selectable marker. (C) IFA of NcLiv*ΔhΔk* parasites expressing Tir1^FLAG^. As expected, Tir1 localizes to the cytoplasm of the parasites. IMC12 is used to show the periphery of the parasites.

We additionally wanted to utilize the NcLivΔ*h*Δ*k* parasites for the study of essential genes; thus, we modified the strain with an auxin-inducible degron (AID) system for conditional protein depletion (Fig. 5A) (31). To do this, we first attempted to express the Tir1 protein using a previously described T. gondii construct (31), but were unable to obtain stably expressing parasites (not shown). We thus replaced the promoter with the NcGRA7 promoter (Fig. 5B), which yielded stable expression of the protein localized to the cytoplasm of the parasite, as expected (Fig. 5C).

### Knockdown of a novel nuclear protein identifies an essential nuclear factor in N. caninum and T. gondii.

**FIG 6:**
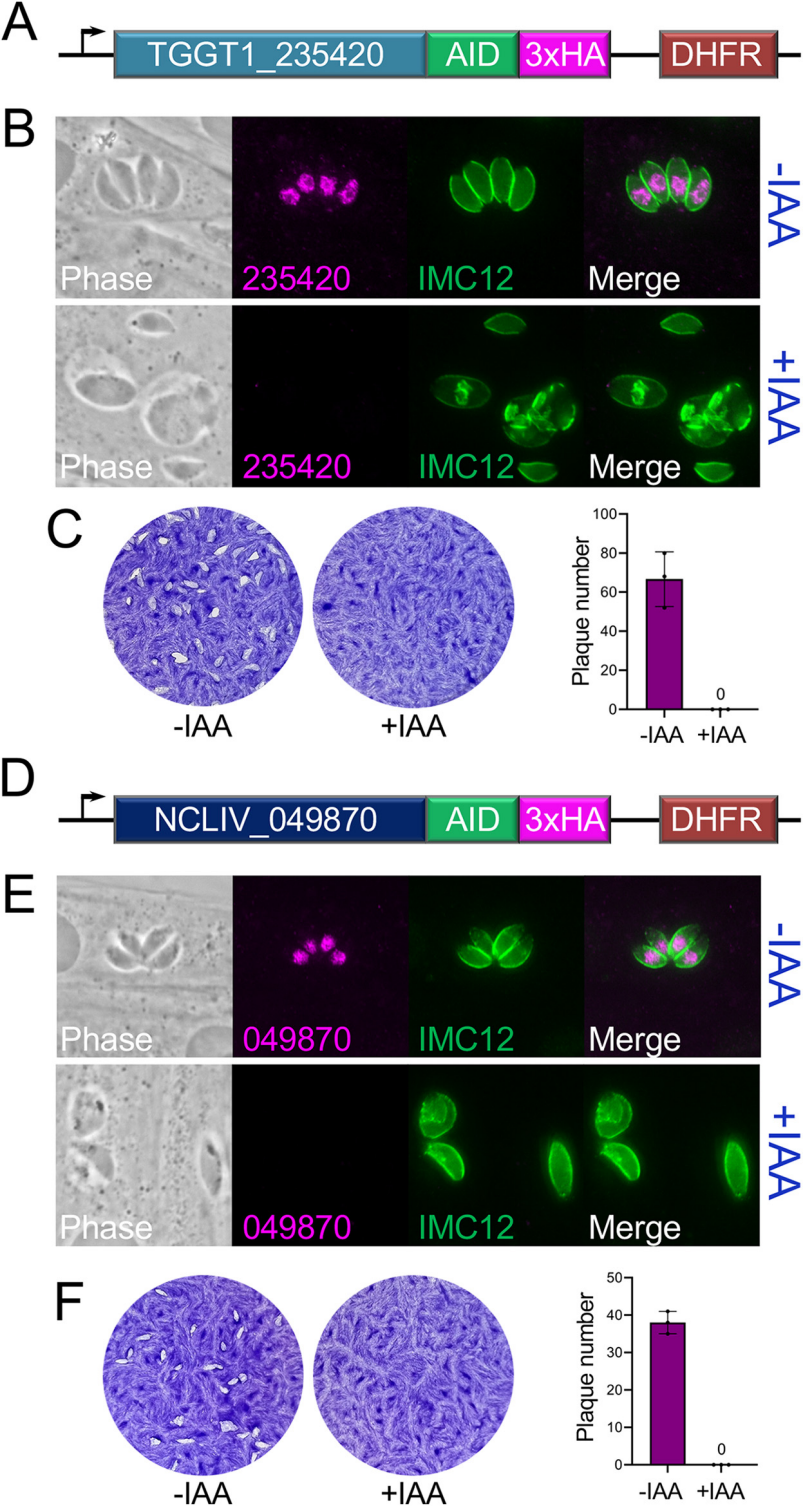
Conditional knockdown of a novel nuclear protein demonstrates essentiality in T. gondii and N. caninum. (A) Diagram of endogenous AID-tagged TGGT1_235420 in Tir1-expressing T. gondii. Tagging includes a 3xHA tag for detection and a *DHFR* cassette for selection. (B) IFA showing that 24-h addition of IAA results in loss of the nuclear HA signal and growth arrest of the parasites. Multiple vacuoles are shown +IAA to highlight that the parasites arrest at a single parasite per vacuole with some in the process of replication. Expanded vacuoles are also observed. (C) Plaque assays and quantification of plaque numbers showing that no plaques are formed upon TGGT1_235420 knockdown, confirming the protein is essential in T. gondii. (D) Diagram of endogenous AID-tagged NCLIV_049870 in Tir1-expressing N. caninum. (E) IFA showing that addition of IAA results in a similar growth arrest of the parasites with swollen vacuoles. (F) Plaque assays and quantification of plaque numbers showing that NCLIV_049870 is essential in N. caninum.

To validate the AID system in N. caninum, we targeted the nuclear protein NCLIV_049870 as we predicted it would be either very important or essential due to the fitness score of its T. gondii orthologue (TGGT1_235420). As a control, we first AID tagged TGGT1_235420 in Tir1-expressing T. gondii and found that TGGT1_235420^AID^ similarly localized to the nucleus (Fig. 6A and B). Knockdown of the protein with indoleacetic acid (IAA) resulted in a dramatic growth arrest of the parasites with the presence of mostly single parasites in a vacuole, some of which were in the process of endodyogeny (Fig. 6B). Many of the parasites also contained additional space within the vacuole. These parasites failed to grow further during elongated IAA treatment, as shown by plaque assays (Fig. 6C), demonstrating that TGGT1_235420 is essential in T. gondii. We then AID tagged NCLIV_049870 in Tir1-expressing N. caninum and found that the AID tagged protein also localized to the nucleus (Fig. 6D and E). IAA treatment of the NCLIV_049870^AID^ parasites resulted in an identical lethal growth arrest with swollen parasites and increased vacuolar space (Fig. 6E and F). This demonstrates the AID system is functional in N. caninum and shows that this newly identified nuclear factor is essential in both parasites.

## DISCUSSION

The close similarity between T. gondii and N. caninum provides an excellent opportunity to explore the factors that are unique to each parasite and are likely to control parasite virulence, their dramatically different host range, and the ability to perform the sexual cycle in distinct definitive hosts. To improve genetic manipulation in N. caninum, we disrupted the NHEJ enzyme Ku80 and found that we were able to obtain highly efficient CRISPR/Cas9-based gene tagging and knockouts. The frequency of tagging and ease of generating full gene knockouts (rather than just insertions) with a guide RNA plus a homology directed repair template are similar to that regularly observed in T. gondii. We additionally exploited the positive and negative selection of *HXGPRT* to generate parasites lacking this selectable marker (in addition to Ku80) for rapid tagging or knockout approaches. Together, these data suggest that recombination in NcLivΔ*h*Δ*k* parasites is similar to that seen in T. gondii and demonstrate the utility of this newly described strain for genetic manipulation in N. caninum.

We also demonstrated that loss of Ku80 results in the dramatic reduction of its dimer partner Ku70, presumably via destabilization and degradation of the protein. In many Δ*ku80* parasites, Ku70 is undetectable, suggesting the *Ku80* knockout is essentially a double knockout of the dimer (which requires both partners for function). This may be surprising as *Ku70* was previously reported to be essential in T. gondii (27). However, these studies were done with early knockout tools that were just under development, and the *Ku70* fitness score (–0.75) in the genome-wide CRISPR/Cas9 screen suggests that it is dispensable (32). A similar degradation of a partner has been seen in the AC9/AC10/Erk7 complex, where knockdown of AC10 results in the complete loss of AC9 from this complex, which is essential for maintenance of the conoid and release of apical secretory organelles (30). This may indicate that T. gondii has a robust system for elimination of misfolded proteins within the parasite, and that ability seems preserved in N. caninum.

Two of the genes that we tagged for localization purposes were secreted proteins. As expected, the *Neospora* GRA48 localized to the vacuole, indicating it is a dense granule protein as is seen in T. gondii, in which it was described to be phosphorylated in the parasitophorous vacuole by another secreted *Toxoplasma* protein, the PV-resident kinase WNG1 (33). Although TgGRA48 has been localized and shown to be activated by another GRA, its function in tachyzoite development and/or host interaction is still unknown. Complementary studies for NcGRA48 may further elucidate the role of GRA48 in parasite biology and enable a comparison between the orthologues of the protein.

The other predicted secreted protein was also demonstrated to be a dense granule protein. We initially selected NSG1 as a target because it was uncharacterized and is *Neospora*-specific, without any known orthologues within the Sarcocystidae family of Apicomplexa. *Neospora* appears to lack a number of the GRAs present in T. gondii that are known to modulate host functions, including GRA15, GRA24, TEEGR, TgIST, and TgNSM (34–39). Those *Toxoplasma* GRAs that are missing in *Neospora* may be important molecules controlling parasite virulence or host range. However, NSG1 is the first *Neospora* GRA that is absent in T. gondii, suggesting that this effector protein may modulate *Neospora*-specific activities in the host or carry out some other function in the *Neospora* parasite vacuole.

The mature NSG1 protein is largely composed of tandem repeats, which are also present in several secreted *Toxoplasma* proteins (e.g., GRA16, MAG2, ROP1, TLN1). While these repeats differ in their sequences and are typically of unknown function, they have been hypothesized to have a function in host cell invasion, immune evasion, and ultimately virulence (40–42). It will be particularly interesting to assess whether there is a strong immune response to NSG1, as this may indicate a role on immunity. In addition, NSG1 may serve as a unique antigen for diagnostic purposes, which have largely centered on the detection of surface antigens or secreted proteins present in both species that have diverged substantially to generate parasite-specific responses (43–45). In that sense, it is remarkable that NSG1 is predicted to have linear B cell epitopes throughout its whole sequence, with high scores for hydrophilicity, accessibility, and flexibility exactly in the region where one of the sets of the tandem repeats are concentrated (Fig. S1B). These features have been well characterized for that purpose in other protozoan parasites such as *Leishmania* and T. cruzi (46, 47).

We additionally modified the NcLivΔ*h*Δ*k* strain with the AID system for the rapid analysis of essential genes. We initially expressed Tir1 using the T. gondii construct that is driven from the tubulin promoter, but the parasites had variable levels of expression, even within parasites in a single vacuole. This variable expression from *Toxoplasma* promoters in *Neospora* has been observed previously in the study of rhoptry proteins (9), and was resolved by using the *Neospora* GRA7 promoter. The variable expression suggests that it may be best practice to use *Neospora* promoters whenever possible, although *Toxoplasma* promoters have been shown to be successful for many uses (9, 10, 20) The AID system in the Δ*ku80* background thus provides a new instrument for rapid conditional knockdown at the protein level in N. caninum.

This usefulness was proven during the characterization of NCLIV_049870 and its orthologue TGGT1_235420. Although yet to be thoroughly characterized, the nuclear proteins in both parasites are essential for parasite growth inside the vacuole. An initial analysis of these genes shows orthologues in other species within the family, and the predicted amino acid sequence shares 60% of identity between T. gondii and N. caninum. While there are no definitive conserved motifs in the proteins that would denote a specific function, analysis of the sequence hints to its nuclear role, probably chromatin-remodeling related. Evolutionary analysis by OrthoMCL indicates that both proteins originated from the ancestor *Sphaeroforma arctica*, which contains a helicase-SANT-associated (HSA) domain that acts as chromatin remodelers by binding nuclear actin-related proteins (48). Although this domain is not preserved in T. gondii, N. caninum, and the closest phylogenetic species, orthologues in Cyclospora cayetanensis, Plasmodium spp and Cryptosporidium spp still contain the HSA domain. In addition, NCLIV_049870 is annotated as “GH22120, related” in ToxoDB, referring to a *Drosophila grimshawi* protein with repeated mucin-like domains. Human proteins containing such domains as MUC-1C are also involved in chromatin remodeling and are indicators of cancer progression and poor clinical outcome (49, 50). Finally, String database prediction for functional partners of these proteins are all chromatin-related: TGME49_267800 (DRPA), TGME49_203950 (BDP), TGME49_289730 (Pep3/Vps18/deep orange family).

In conclusion, it is well known that molecular genetics provides powerful investigative approaches that can greatly improve our understanding of intracellular pathogens and their interactions with host cells. This study dramatically improves the ability for genetic manipulation of N. caninum, similar to the tools available in T. gondii. The development of molecular genetics for N. caninum will enhance knowledge of neosporosis, enable a better comparison of N. caninum and T. gondii, and provide an efficient heterologous expression system to unravel the processing, targeting, and function of T. gondii proteins.

## MATERIALS AND METHODS

### Host cells and parasite culture.

Parental NcLiv and modified strains of N. caninum, as well as RHΔ*hxgprt*Δ*ku80* and modified strains of T. gondii, were maintained on confluent monolayers of human foreskin fibroblast (HFF) cells, cultured in complete Dulbecco's Modified Eagle's Medium (DMEM) supplemented with 5% Fetal Bovine Serum (Gibco), 5% Cosmic Calf Serum (HyClone), 2 mM glutamine, 100 U/mL penicillin, and 100 μg/mL streptomycin, at 37°C in 5% CO_2_ atmosphere, as previously described (51, 52).

### *In silico* analysis of POI.

All general information on proteins of interest (POIs) such as sequences, essentiality, genomic location, orthology profile, among others, were obtained at ToxoDB (www.toxodb.org). Orthologous grouping of evolutive and expanded families of POIs were predicted by OrthoMCL (orthomcl.org) and Eukaryotic Pathogen, Vector and Host Informatics Resource (VEuPathDB, veupathdb.org), based on amino acid sequences. We used SignalP 6.0 (services.healthtech.dtu.dk/service.php?SignalP-6.0) for the prediction of signal peptides and cleavage sites of the POI sequences and TMHMM Server v. 2.0 (http://www.cbs.dtu.dk/services/TMHMM/) for the prediction of transmembrane helices. Prediction of structure and/or function of the POIs was performed on the Protein Homology/analogy Recognition Engine V 2.0 (PHYRE2, www.sbg.bio.ic.ac.uk/phyre2). For detection of tandem repeats in the studied POI, we used Rapid Automatic Detection and Alignment of Repeats in Protein Sequences (RADAR, www.ebi.ac.uk/Tools/pfa/radar), along with manual curation of the data. B cell immunogenicity and linear epitope predictions were obtained at Immune Epitope Database and Analysis Resource (IEDB, www.iedb.org), through the following tools: Bepipred Linear Epitope Prediction 2.0 (53); Parker Hydrophilicity Prediction (54); Emini Surface Accessibility Prediction (55); and Karplus & Schulz Flexibility Prediction (56). String (string-db.org) was used for the prediction of potential protein interactions and functional enrichment analysis.

### Epitope tagging.

We used CRISPR/Cas9 for endogenous tagging of genes of interest—NCLIV_056045/*Nc*Ku80 (primers P1-P4), NCLIV_064150/*Nc*Ku70 (primers P5-P8), NCLIV_069360/*NcGRA48* (primers P9-P12), NCLIV_066730/*NSG1* (primers P13-P16), NCLIV_049870 (primers P17-P20)—with 3xHA, as previously described (29). Oligonucleotides used for this and other purposes are listed in Table S1. Briefly, sequences encoding gRNAs were ligated into the pU6 Universal plasmid and prepared along with a PCR product from the *pLIC:3xHA:DHFR* vector with 40 bp flanking regions for recombination at the 3′ end of each gene. Transfection was performed in 2 mm gap cuvettes with 400 μL of cytomix containing 10^7^ freshly lysed tachyzoites, 100 μg of each construct, and CaCl_2_ (150 μM), using single pulses of 1.5–2.0 ms at 1.5 kV, 25 Ω and 25 μF in an electroporator (Bio-Rad), as previously described (19). Parasites were then selected in medium containing 1 μM pyrimethamine and cloned by limiting dilution. Tagged clones were screened by IFA using the HA.11 monoclonal antibody (BioLegend). Cross-reactive rabbit antibodies against *Toxoplasma* IMC12 (Back et al., unpublished data) were used to stain the periphery of the parasites. Epitope tagging efficiency was calculated by counting the HA positive vacuoles versus IMC12 positive vacuoles at 14 days posttransfection. At least 100 vacuoles per strain were counted to determine the percentage of parasites successfully tagged.

### Gene disruption.

CRISPR/Cas9 and homologous recombination were used to disrupt selected targets, as previously described (29). For *NcHXGPRT* (NcLivΔ*hxgprt*), a single sgRNA (primers P21/P22) was used to disrupt the GOI’s coding region. For *NcKu80* (NcLivΔ*h*Δ*k*), flanking sequences from the 5’UTR (forward, 559 bp) and 3’UTR (reverse, 528 bp) were amplified from N. caninum genomic DNA (primers P23-P26), inserted into a cassette containing T. gondii *HXGPRT* driven by the N. caninum *GRA7* promoter (pJET1.2:p*NcGRA7*:*TgHXGPRT*), and transfected into NcLivΔ*hxgprt* parasites with a gRNA (primers P27/P28) directed to the beta-barrel domain of the gene. To remove the selectable marker, NcLivΔ*h*Δ*k* parasites were transfected with a gRNA (primers P29/P30) directed to the *TgHXGPRT* coding region and an AccI truncated version of the original cassette as a repair template (–1633 bp). This eliminated most of the *NcGRA7* promoter and *TgHXGPRT* coding region, although retaining approximately 2000 bp for efficient homologous recombination. To generate NcLivΔ069360/*NcGRA48* and NcLivΔ066730/*NSG1* strains, epitope tagged NcLivΔ*h*Δ*k* parasites were transfected with sgRNA directed to the GOI’s coding regions, along with a PCR product from the *pJET1.2:pNcGRA7:TgHXGPRT* with 39 bp homology sequences of its UTR regions (*NcGRA48*: primers P31-P34; *NSG1*: primers P35-P38).

### Auxin-inducible degron modifications to N. caninum.

To produce N. caninum stably expressing the transport inhibitor response 1 (Tir1) auxin receptor from Oryza sativa, the original plasmids generated and codon optimized for T. gondii (*pTgTUB1:OsTIR1-3xFLAG*, *TgSAG1:CAT*) (31) were adapted to the N. caninum system by Gibson assembly. Briefly, the *Toxoplasma TUB1* promoter was replaced by the *Neospora GRA7* promoter (pNcGRA7, primers P39-P42). In addition, the selectable marker used was also changed to *TgHXGPRT* instead of *chloramphenicol acetyltransferase* (*CAT*) used in the original description (primers P43-P46). These changes generated the plasmid *pNcGRA7:OsTIR1-3xFLAG*, *TgSAG1:TgHXGPRT*, used in this work. NcLivΔ*h*Δ*k* parasites were transfected with this linearized construct, selected with Mpa/X, cloned by limiting dilution, and a clone was chosen with strong cytoplasmic staining with the M2 anti-Flag Mab (Sigma). The original *pTgTUB1:YFP-mAID-3xHA*, *HXGPRT* plasmid, containing the mAID sequence for proteasomal degradation, was altered by changing its selectable marker to *DHFR*, generating the *pTgTUB1:YFP-mAID-3xHA*, *DHFR* plasmid (31). For depletion of AID fusion proteins, the parasites were treated with 500 μM indoleacetic acid (IAA) added at the time of infection for the specified time periods.

### Plaque assays.

Six-well plates were seeded with confluent monolayers of HFFs and infected with approximately 250 parasites (±500 μM IAA) and allowed to form plaques for 6 days for T. gondii and 8 days for N. caninum. The monolayers were then fixed in 100% methanol for 3 min, washed with phosphate-buffered saline (PBS), and stained with crystal violet for visualization. The number of plaques were quantified from the samples and the plaque assays were performed in triplicate.

### Immunofluorescence assay and fluorescence microscopy.

For IFAs, tachyzoites were used to infect coverslips of HFFs for 24 h. The coverslips were then fixed in 3.7% formaldehyde/PBS for 15 min. The coverslips were washed in phosphate-buffered saline (PBS) and blocked and permeabilized in a solution containing PBS/3% bovine serum albumin (BSA)/0.2% Triton X-100 for 30 min. Samples were then incubated with mouse monoclonal anti-HA antibodies (HA.11, BioLegend) and rabbit anti-IMC12 (Back et al., unpublished data) diluted in PBS, along with BSA (3%) and Triton X-100 (0.2%) for 1 h. The samples were then washed in PBS and treated with species-specific secondary antibodies conjugated to Alexa 594/488, and diluted 1:2000 in PBS/3%BSA/0.2%TX-100 Hoechst staining was performed using Hoechst 33342 (5 μg/mL) in PBS for 5 min. Following a new washing cycle, coverslips were mounted onto microscope slides with Vectashield (Vector Labs) mounting media, and the fluorescence was observed using a Zeiss Axio Imager Z1 microscope. Images were processed via deconvolution using the Zeiss Zen software.

### Western blot analysis.

Whole-parasite lysates were separated by 10% SDS-PAGE. Samples were transferred to nitrocellulose overnight and probed with primary antibodies. For all secondary antibody incubations, horseradish peroxidase (HRP)-conjugated goat anti-mouse or goat anti-rabbit antibodies were used at a 1:2000 dilution. Following secondary incubation, a chemiluminescent substrate was used for the detection of HRP activity.

10.1128/msphere.00896-21.1 FIG S1 Features of NCLIV_066730/NSG-1. (A) Amino acid sequence of NCLIV_066730/NSG1 highlighting with the predicted signal peptide (green) as well as a series of repeats (pink and red) in the mature protein. Repeats were identified using the RADAR protein repeat finder and curated manually. (B) The presence of antibody binding sites in the amino acid sequence of NCLIV_066730/NSG1 was predicted using four different algorithms at the Immune Epitope Database and Analysis Resource (IEDB), as follows: Bepipred Linear Epitope Prediction 2.0; Parker Hydrophilicity Prediction; Emini Surface Accessibility Prediction; and Karplus & Schulz Flexibility Prediction. Download FIG S1, TIF file, 1.0 MB. Copyright © 2022 Mineo et al. 2022 Mineo et al. https://creativecommons.org/licenses/by/4.0/ This content is distributed under the terms of the Creative Commons Attribution 4.0 International license.

10.1128/msphere.00896-21.2 TABLE S1 Oligonucleotide primers used in this study. All primer sequences are shown in the 5′ to 3′ orientation. Download Table S1, PDF file, 0.02 MB. Copyright © 2022 Mineo et al. 2022 Mineo et al. https://creativecommons.org/licenses/by/4.0/ This content is distributed under the terms of the Creative Commons Attribution 4.0 International license.

## References

[1] Smith NC, Goulart C, Hayward JA, Kupz A, Miller CM, van Dooren GG. 2021. Control of human toxoplasmosis. Int J Parasitol 51:95–121. doi:10.1016/j.ijpara.2020.11.001. 33347832

[2] Lindsay DS, Dubey JP. 2020. Neosporosis, toxoplasmosis, and sarcocystosis in ruminants: an update. Vet Clin North Am Food Anim Pract 36:205–222. doi:10.1016/j.cvfa.2019.11.004. 32029185

[3] Reichel MP, Alejandra Ayanegui-Alcérreca M, Gondim LFP, Ellis JT. 2013. What is the global economic impact of *Neospora caninum* in cattle—the billion dollar question. Int J Parasitol 43:133–142. doi:10.1016/j.ijpara.2012.10.022. 23246675

[4] Soete M, Hettman C, Soldati D. 1999. The importance of reverse genetics in determining gene function in apicomplexan parasites. Parasitology 118:53–61. doi:10.1017/s003118209900414x. 10466137

[5] Di Cristina M, Carruthers VB. 2018. New and emerging uses of CRISPR/Cas9 to genetically manipulate apicomplexan parasites. Parasitology 145:1119–1126. doi:10.1017/S003118201800001X. 29463318 PMC6063771

[6] Boothroyd JC. 2020. What a difference 30 years makes! A perspective on changes in research methodologies used to study *Toxoplasma gondii*. Methods Mol Biol 2071:1–25. doi:10.1007/978-1-4939-9857-9_1. 31758444

[7] Howe DK, Mercier C, Messina M, Sibley LD. 1997. Expression of *Toxoplasma gondii* genes in the closely-related apicomplexan parasite *Neospora caninum*. Mol Biochem Parasitol 86:29–36. doi:10.1016/S0166-6851(97)90003-7. 9178265

[8] Howe DK, Sibley LD. 1997. Development of molecular genetics for *Neospora caninum*: a complementary system to *Toxoplasma gondii*. Methods 13:123–133. doi:10.1006/meth.1997.0505. 9405196

[9] Beckers CJ, Wakefield T, Joiner KA. 1997. The expression of *Toxoplasma* proteins in *Neospora caninum* and the identification of a gene encoding a novel rhoptry protein. Mol Biochem Parasitol 89:209–223. doi:10.1016/s0166-6851(97)00120-5. 9364966

[10] Zhang G, Huang X, Boldbaatar D, Battur B, Battsetseg B, Zhang H, Yu L, Li Y, Luo Y, Cao S, Goo Y-K, Yamagishi J, Zhou J, Zhang S, Suzuki H, Igarashi I, Mikami T, Nishikawa Y, Xuan X. 2010. Construction of *Neospora caninum* stably expressing TgSAG1 and evaluation of its protective effects against *Toxoplasma gondii* infection in mice. Vaccine 28:7243–7247. doi:10.1016/j.vaccine.2010.08.096. 20832493

[11] Lei T, Wang H, Liu J, Nan H, Liu Q. 2014. ROP18 is a key factor responsible for virulence difference between *Toxoplasma gondii* and *Neospora caninum*. PLoS One 9:e99744. doi:10.1371/journal.pone.0099744. 24927100 PMC4057265

[12] Franco M, Shastri AJ, Boothroyd JC. 2014. Infection by *Toxoplasma gondii* specifically induces host c-Myc and the genes this pivotal transcription factor regulates. Eukaryot Cell 13:483–493. doi:10.1128/EC.00316-13. 24532536 PMC4000098

[13] Franco M, Panas MW, Marino ND, Lee M-CW, Buchholz KR, Kelly FD, Bednarski JJ, Sleckman BP, Pourmand N, Boothroyd JC. 2016. A novel secreted protein, MYR1, is central to *Toxoplasma*’s manipulation of host cells. mBio 7:e02231-15. doi:10.1128/mBio.02231-15. 26838724 PMC4742717

[14] Collantes-Fernandez E, Arrighi RBG, Alvarez-García G, Weidner JM, Regidor-Cerrillo J, Boothroyd JC, Ortega-Mora LM, Barragan A. 2012. Infected dendritic cells facilitate systemic dissemination and transplacental passage of the obligate intracellular parasite *Neospora caninum* in mice. PLoS One 7:e32123. doi:10.1371/journal.pone.0032123. 22403627 PMC3293873

[15] Donald RG, Carter D, Ullman B, Roos DS. 1996. Insertional tagging, cloning, and expression of the *Toxoplasma gondii* hypoxanthine-xanthine-guanine phosphoribosyltransferase gene: use as a selectable marker for stable transformation. J Biol Chem 271:14010–14019. doi:10.1074/jbc.271.24.14010. 8662859

[16] Pereira LM, Baroni L, Yatsuda AP. 2014. A transgenic *Neospora caninum* strain based on mutations of the dihydrofolate reductase-thymidylate synthase gene. Exp Parasitol 138:40–47. doi:10.1016/j.exppara.2014.01.004. 24440296

[17] Pereira LM, Yatsuda AP. 2014. The chloramphenicol acetyltransferase vector as a tool for stable tagging of *Neospora caninum*. Mol Biochem Parasitol 196:75–81. doi:10.1016/j.molbiopara.2014.08.001. 25127750

[18] Shen B, Brown KM, Lee TD, Sibley LD. 2014. Efficient gene disruption in diverse strains of *Toxoplasma gondii* using CRISPR/CAS9. mBio 5:e01114-14. doi:10.1128/mBio.01114-14. 24825012 PMC4030483

[19] Sidik SM, Hackett CG, Tran F, Westwood NJ, Lourido S. 2014. Efficient genome engineering of *Toxoplasma gondii* using CRISPR/Cas9. PLoS One 9:e100450. doi:10.1371/journal.pone.0100450. 24971596 PMC4074098

[20] Arranz-Solís D, Regidor-Cerrillo J, Lourido S, Ortega-Mora LM, Saeij JPJ. 2018. Toxoplasma CRISPR/Cas9 constructs are functional for gene disruption in *Neospora caninum*. Int J Parasitol 48:597–600. doi:10.1016/j.ijpara.2018.03.002. 29625127 PMC7025762

[21] Yang C, Liu J, Ma L, Zhang X, Zhang X, Zhou B, Zhu X, Liu Q. 2018. NcGRA17 is an important regulator of parasitophorous vacuole morphology and pathogenicity of *Neospora caninum*. Vet Parasitol 264:26–34. doi:10.1016/j.vetpar.2018.03.018. 30503087

[22] Nishikawa Y, Shimoda N, Fereig RM, Moritaka T, Umeda K, Nishimura M, Ihara F, Kobayashi K, Himori Y, Suzuki Y, Furuoka H. 2018. *Neospora caninum* dense granule protein 7 regulates the pathogenesis of neosporosis by modulating host immune response. Appl Environ Microbiol 84:e01350-18. doi:10.1128/AEM.01350-18. 30006392 PMC6121998

[23] Wang F, Wang X, Song X, Ma L, Yang J, Liu Q, Liu J. 2021. Function of *Neospora caninum* dense granule protein 7 in innate immunity in mice. Parasitol Res 120:197–207. doi:10.1007/s00436-020-06961-4. 33164154

[24] Dong J, Zhang N, Zhao P, Li J, Cao L, Wang X, Li X, Yang J, Zhang X, Gong P. 2021. Disruption of dense granular protein 2 (GRA2) decreases the virulence of *Neospora caninum*. Front Vet Sci 8:634612. doi:10.3389/fvets.2021.634612. 33681332 PMC7933011

[25] Wang C, Yang C, Liu J, Liu Q. 2020. NcPuf1 Is a key virulence factor in *Neospora caninum*. Pathogens 9:1019. doi:10.3390/pathogens9121019. PMC7761618 33276672

[26] Huynh M-H, Carruthers VB. 2009. Tagging of endogenous genes in a *Toxoplasma gondii* strain lacking Ku80. Eukaryot Cell 8:530–539. doi:10.1128/EC.00358-08. 19218426 PMC2669203

[27] Fox BA, Ristuccia JG, Gigley JP, Bzik DJ. 2009. Efficient gene replacements in *Toxoplasma gondii* strains deficient for nonhomologous end joining. Eukaryot Cell 8:520–529. doi:10.1128/EC.00357-08. 19218423 PMC2669201

[28] Walker JR, Corpina RA, Goldberg J. 2001. Structure of the Ku heterodimer bound to DNA and its implications for double-strand break repair. Nature 412:607–614. doi:10.1038/35088000. 11493912

[29] Nadipuram SM, Thind AC, Rayatpisheh S, Wohlschlegel JA, Bradley PJ. 2020. Proximity biotinylation reveals novel secreted dense granule proteins of *Toxoplasma gondii* bradyzoites. PLoS One 15:e0232552. doi:10.1371/journal.pone.0232552. 32374791 PMC7202600

[30] Back PS, O'Shaughnessy WJ, Moon AS, Dewangan PS, Hu X, Sha J, Wohlschlegel JA, Bradley PJ, Reese ML. 2020. Ancient MAPK ERK7 is regulated by an unusual inhibitory scaffold required for *Toxoplasma* apical complex biogenesis. Proc Natl Acad Sci USA 117:12164–12173. doi:10.1073/pnas.1921245117. 32409604 PMC7275706

[31] Brown KM, Long S, Sibley LD. 2018. Conditional knockdown of proteins using auxin-inducible degron (AID) fusions in *Toxoplasma gondii*. Bio Protoc 8:e2728. 10.21769/BioProtoc.2728 PMC5890294 29644255

[32] Sidik SM, Huet D, Ganesan SM, Huynh M-H, Wang T, Nasamu AS, Thiru P, Saeij JPJ, Carruthers VB, Niles JC, Lourido S. 2016. A genome-wide CRISPR screen in *Toxoplasma* identifies essential apicomplexan genes. Cell 166:1423–1435.e12. doi:10.1016/j.cell.2016.08.019. 27594426 PMC5017925

[33] Beraki T, Hu X, Broncel M, Young JC, O'Shaughnessy WJ, Borek D, Treeck M, Reese ML. 2019. Divergent kinase regulates membrane ultrastructure of the *Toxoplasma* parasitophorous vacuole. Proc Natl Acad Sci USA 116:6361–6370. doi:10.1073/pnas.1816161116. 30850550 PMC6442604

[34] Sangaré LO, Yang N, Konstantinou EK, Lu D, Mukhopadhyay D, Young LH, Saeij JPJ. 2019. Toxoplasma GRA15 activates the NF-κB Pathway through Interactions with TNF receptor-associated factors. mBio 10:e00808-19. doi:10.1128/mBio.00808-19. 31311877 PMC6635525

[35] Mukhopadhyay D, Arranz-Solís D, Saeij JPJ. 2020. Toxoplasma GRA15 and GRA24 are important activators of the host innate immune response in the absence of TLR11. PLoS Pathog 16:e1008586. doi:10.1371/journal.ppat.1008586. 32453782 PMC7274473

[36] Braun L, Brenier-Pinchart M-P, Yogavel M, Curt-Varesano A, Curt-Bertini R-L, Hussain T, Kieffer-Jaquinod S, Coute Y, Pelloux H, Tardieux I, Sharma A, Belrhali H, Bougdour A, Hakimi M-A. 2013. A *Toxoplasma* dense granule protein, GRA24, modulates the early immune response to infection by promoting a direct and sustained host p38 MAPK activation. J Exp Med 210:2071–2086. doi:10.1084/jem.20130103. 24043761 PMC3782045

[37] Olias P, Etheridge RD, Zhang Y, Holtzman MJ, Sibley LD. 2016. Toxoplasma effector recruits the Mi-2/NuRD complex to repress STAT1 transcription and block IFN-γ-dependent gene expression. Cell Host Microbe 20:72–82. doi:10.1016/j.chom.2016.06.006. 27414498 PMC4947229

[38] Gay G, Braun L, Brenier-Pinchart M-P, Vollaire J, Josserand V, Bertini R-L, Varesano A, Touquet B, De Bock P-J, Coute Y, Tardieux I, Bougdour A, Hakimi M-A. 2016. *Toxoplasma gondii* TgIST co-opts host chromatin repressors dampening STAT1-dependent gene regulation and IFN-γ-mediated host defenses. J Exp Med 213:1779–1798. doi:10.1084/jem.20160340. 27503074 PMC4995087

[39] Rosenberg A, Sibley LD. 2021. *Toxoplasma gondii* secreted effectors co-opt host repressor complexes to inhibit necroptosis. Cell Host Microbe 29:1186–1198.e8. doi:10.1016/j.chom.2021.04.016. 34043960 PMC8711274

[40] Liew FY, Millott SM, Schmidt JA. 1990. A repetitive peptide of Leishmania can activate T helper type 2 cells and enhance disease progression. J Exp Med 172:1359–1365. doi:10.1084/jem.172.5.1359. 2146362 PMC2188667

[41] Stahl HD, Crewther PE, Anders RF, Brown GV, Coppel RL, Bianco AE, Mitchell GF, Kemp DJ. 1985. Interspersed blocks of repetitive and charged amino acids in a dominant immunogen of *Plasmodium falciparum*. Proc Natl Acad Sci USA 82:543–547. doi:10.1073/pnas.82.2.543. 3881769 PMC397076

[42] Gao W, Wortis HH, Pereira MA. 2002. The *Trypanosoma cruzi* trans-sialidase is a T cell-independent B cell mitogen and an inducer of non-specific Ig secretion. Int Immunol 14:299–308. doi:10.1093/intimm/14.3.299. 11867566

[43] Pinheiro AF, Borsuk S, Berne MEA, Pinto L da S, Andreotti R, Roos T, Rollof BC, Leite FPL. 2013. Expression of *Neospora caninum* NcSRS2 surface protein in *Pichia pastoris* and its application for serodiagnosis of *Neospora* infection. Pathog Glob Health 107:116–121. doi:10.1179/2047773213Y.0000000082. 23683365 PMC4003588

[44] Ybañez RHD, Terkawi MA, Kameyama K, Xuan X, Nishikawa Y. 2013. Identification of a highly antigenic region of subtilisin-like serine protease 1 for serodiagnosis of *Neospora caninum* infection. Clin Vaccine Immunol 20:1617–1622. doi:10.1128/CVI.00352-13. 23966554 PMC3807189

[45] Pereira HS, E Almeida LT, Fernandes V, Senra RL, Fontes PP, Bittar ER, Ribon A, de OB, Rotta PP, Menezes-Souza D, Bittar JFF, de Mendes TAO. 2020. Chimeric protein designed by genome-scale immunoinformatics enhances serodiagnosis of bovine neosporosis. J Clin Microbiol 58:e01343-19. doi:10.1128/JCM.01343-19. 32404479 PMC7315025

[46] Goto Y, Carter D, Reed SG. 2008. Immunological dominance of *Trypanosoma cruzi* tandem repeat proteins. Infect Immun 76:3967–3974. doi:10.1128/IAI.00604-08. 18625739 PMC2519453

[47] Goto Y, Carter D, Guderian J, Inoue N, Kawazu S-I, Reed SG. 2010. Upregulated expression of B-cell antigen family tandem repeat proteins by Leishmania amastigotes. Infect Immun 78:2138–2145. doi:10.1128/IAI.01102-09. 20160013 PMC2863543

[48] Szerlong H, Hinata K, Viswanathan R, Erdjument-Bromage H, Tempst P, Cairns BR. 2008. The HSA domain binds nuclear actin-related proteins to regulate chromatin-remodeling ATPases. Nat Struct Mol Biol 15:469–476. doi:10.1038/nsmb.1403. 18408732 PMC2810487

[49] Hata T, Rajabi H, Takahashi H, Yasumizu Y, Li W, Jin C, Long MD, Hu Q, Liu S, Fushimi A, Yamashita N, Kui L, Hong D, Yamamoto M, Miyo M, Hiraki M, Maeda T, Suzuki Y, Samur MK, Kufe D. 2019. MUC1-C activates the NuRD complex to drive dedifferentiation of triple-negative breast cancer cells. Cancer Res 79:5711–5722. doi:10.1158/0008-5472.CAN-19-1034. 31519689 PMC6881519

[50] Yamamoto M, Jin C, Hata T, Yasumizu Y, Zhang Y, Hong D, Maeda T, Miyo M, Hiraki M, Suzuki Y, Hinohara K, Rajabi H, Kufe D. 2019. MUC1-C integrates chromatin remodeling and PARP1 activity in the DNA damage response of triple-negative breast cancer cells. Cancer Res 79:2031–2041. doi:10.1158/0008-5472.CAN-18-3259. 30824588 PMC6467768

[51] Donald RG, Roos DS. 1993. Stable molecular transformation of *Toxoplasma gondii*: a selectable dihydrofolate reductase-thymidylate synthase marker based on drug-resistance mutations in malaria. Proc Natl Acad Sci USA 90:11703–11707. doi:10.1073/pnas.90.24.11703. 8265612 PMC48052

[52] Sohn CS, Cheng TT, Drummond ML, Peng ED, Vermont SJ, Xia D, Cheng SJ, Wastling JM, Bradley PJ. 2011. Identification of novel proteins in *Neospora caninum* using an organelle purification and monoclonal antibody approach. PLoS One 6:e18383. doi:10.1371/journal.pone.0018383. 21483743 PMC3070720

[53] Jespersen MC, Peters B, Nielsen M, Marcatili P. 2017. BepiPred-2.0: improving sequence-based B-cell epitope prediction using conformational epitopes. Nucleic Acids Res 45:W24–W29. doi:10.1093/nar/gkx346. 28472356 PMC5570230

[54] Parker JM, Guo D, Hodges RS. 1986. New hydrophilicity scale derived from high-performance liquid chromatography peptide retention data: correlation of predicted surface residues with antigenicity and X-ray-derived accessible sites. Biochemistry 25:5425–5432. doi:10.1021/bi00367a013. 2430611

[55] Emini EA, Hughes JV, Perlow DS, Boger J. 1985. Induction of hepatitis A virus-neutralizing antibody by a virus-specific synthetic peptide. J Virol 55:836–839. doi:10.1128/JVI.55.3.836-839.1985. 2991600 PMC255070

[56] Karplus PA, Schulz GE. 1985. Prediction of chain flexibility in proteins. Naturwissenschaften 72:212–213. doi:10.1007/BF01195768.

