# Supplementary figures and images for: Efficient Gene Knockout and Knockdown Systems in Neospora caninum Enable Rapid Discovery and Functional Assessment of Novel Proteins
Source: mSphere. 2022 Jan 12;7(1):e00896-21. doi: 10.1128/msphere.00896-21 (PMC8754167; doi:10.1128/msphere.00896-21)

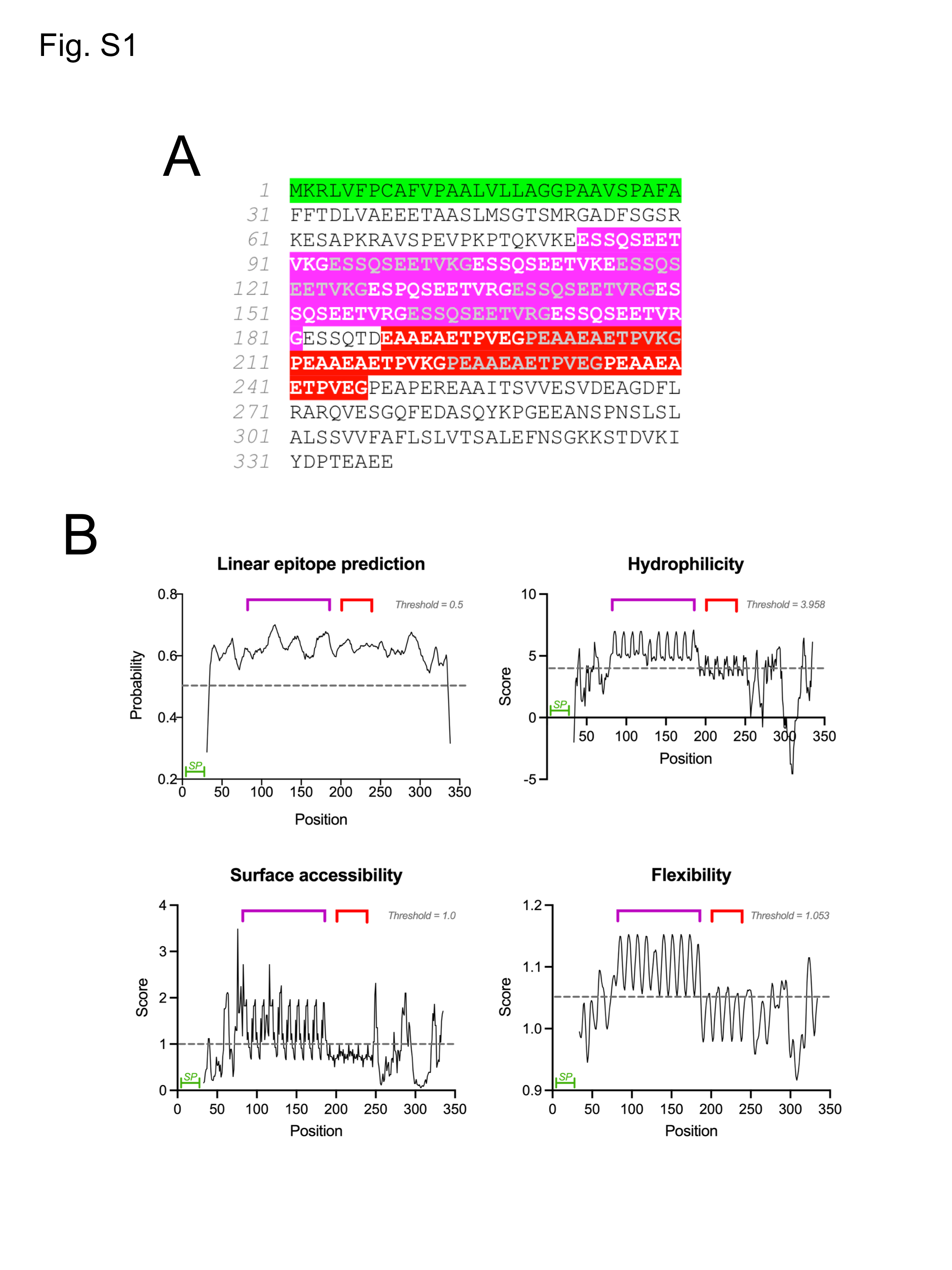

Supplement: FIG S1 [file msphere.00896-21-sf001.tif]
