# Supplementary material for: Efficient Gene Knockout and Knockdown Systems in Neospora caninum Enable Rapid Discovery and Functional Assessment of Novel Proteins
Source: mSphere. 2022 Jan 12;7(1):e00896-21. doi: 10.1128/msphere.00896-21 (PMC8754167; doi:10.1128/msphere.00896-21)
Supplement: TABLE S1 [file msphere.00896-21-st001.pdf]

Table S1. Oligonucleotides used in this study.

Primers

Name	Description	Sequence (5'-3')
P1	NCLIV_056045 tag homology flanks FWD	AGATCCGGTTGATGTTGATGATGTGCTCGATCTTGTGGAAGGAAGTGGAGGACGGGAATT
P2	NCLIV_056045 tag homology flanks REV	TCAATTAGGCGAGCCTTCTTAGTCAGCGTGGCCAACTGCGACGGCCAGTGAATTGTAATA
P3	NCLIV_056045 tag gRNA FWD	AAGTTGGTCGAGTCGAAAATACCCCAG
P4	NCLIV_056045 tag gRNA REV	AAAACTGGGGTATTTTCGACTCGACCA
P5	NCLIV_064150 tag homology flanks FWD	TTTAGTCCAGCAAGCAGAAACCATTTGGGAAAGCGAGAAGGGAAGTGGAGGACGGGAATT
P6	NCLIV_064150 tag homology flanks REV	ATCCATAACGCGCCATGTAAACCTGCGCAACGGCATACCGACGGCCAGTGAATTGTAATA
P7	NCLIV_064150 tag gRNA FWD	AAGTTGCGTCTGCAACCCAATAGATG
P8	NCLIV_064150 tag gRNA REV	AAAACATCTATTGGGTTGCAGACGCA
P9	NCLIV_069360 tag homology flanks FWD	ACCCGTGACCAATGTGGAGAACGCAGTCCCACTGGGGAAGGGAAGTGGAGGACGGGAATT
P10	NCLIV_069360 tag homology flanks REV	AGTCCTTTTCCAATGATGGAACGGACTCGCGCGTTTGTCGACGGCCAGTGAATTGTAATA
P11	NCLIV_069360 tag gRNA FWD	AAGTTGCAATTCGGGATGCTGCTGCGG
P12	NCLIV_069360 tag gRNA REV	AAAACCGCAGCAGCATCCCGAATTGCA
P13	NCLIV_066730 tag homology flanks FWD	TACAGACGTCAAAATCTATGACCCCACGGAGGCCGAAGAAGGAAGTGGAGGACGGGAATT
P14	NCLIV_066730 tag homology flanks REV	CTTGTAACTTTGGCGGTTCGCACGTAGATGCTTCCTACCGACGGCCAGTGAATTGTAATA
P15	NCLIV_066730 tag gRNA FWD	AAGTTGTCTGGGCACGGACTTGTGAG
P16	NCLIV_066730 tag gRNA REV	AAAACTCACAAGTCCGTGCCCAGACA
P17	NCLIV_049870 tag homology flanks FWD	CTATCCGGCTCCTGCGCCGGGTCAGTTCGAGGAAGGCGCAGGAAGTGGAGGACGGGAATT
P18	NCLIV_049870 tag homology flanks REV	CAGTTCTCGCGCTACGGCGTTTCCTCCATGCTCTGCCGCGACGGCCAGTGAATTGTAATA
P19	NCLIV_049870 tag gRNA FWD	AAGTTAGAGAACGCGAAAAGGACGTG
P20	NCLIV_049870 tag gRNA REV	AAAACACGTCCTTTTCGCGTTCTCTA
P21	NCLIV_038170 gRNA FWD	AAGTTGATAAGATCCTCCTTCCTGGG
P22	NCLIV_038170 gRNA REV	AAAACCCAGGAAGGAGGATCTTATCA
P23	NCLIV_056045 KO 5'UTR homology flanks for PJET FWD	GCGGCCGCGCATCAGTAACAGACCGATAC
P24	NCLIV_056045 KO 5'UTR homology flanks for PJET REV	GAATTCGCGGAGATATGCACACAAATG
P25	NCLIV_056045 KO 3'UTR homology flanks for PJET FWD	GAATTCGCTACGCGATACCTCTACATA
P26	NCLIV_056045 KO 3'UTR homology flanks for PJET REV	GAGCTCGTTCCACAAACCAGTTAGCCT
P27	NCLIV_056045 KO gRNA FWD	AAGTTGCTTCTGATGAGGTCTTGCGAG
P28	NCLIV_056045 KO gRNA REV	AAAACTCGCAAGACCTCATCAGAAGCA
P29	TgHXGPRT gRNA cleanup FWD	AAGTTGCCTCCCTGGTGGATTGGTCAG
P30	TgHXGPRT gRNA cleanup REV	AAAACTGACCAATCCACCAGGGAGGCA
P31	NCLIV_069360 KO homology flanks FWD	TACCCTTTCCTTGCCTGTGTAACTTGCTCACACGTCAGCCCACTCCATGGAACCTGACTG
P32	NCLIV_069360 KO homology flanks REV	CACGATGTTCGATGCAAGGGACCGAACAGTCGCCAGATCCCTGCAAGTGCATAGAAGGAA
P33	NCLIV_069360 KO gRNA FWD	AAGTTATAGTTAAGGGGTAGGCCATG
P34	NCLIV_069360 KO gRNA REV	AAAACATGGCCTACCCCTTAACTATA
P35	NCLIV_066730 KO homology flanks FWD	TTTTAGCTGTCACTTCCGAAACTCTCGTTTTCTTCCGCACCACTCCATGGAACCTGACTG
P36	NCLIV_066730 KO homology flanks REV	CATGCCGTTTGTTTCAGAGGACCGCGCGACACAGGAAGGCCTGCAAGTGCATAGAAGGAA
P37	NCLIV_066730 KO gRNA FWD	AAGTTGCAGTCTGAGGAGACAGTCAG
P38	NCLIV_066730 KO gRNA REV	AAAACTGACTGTCTCCTCAGACTGCA
P39	Tir1_vector Gibson for promoter FWD	CGTCTCAGTGTCCTCCTAAAATGACCTACTTCCCTGAG
P40	Tir1_vector Gibson for promoter REV	AAGCTCTAGCTTATCGATAC
P41	pNcGRA7_insert Gibson FWD	GTATCGATAAGCTAGAGCTTCCACTCCATGGAACCTGAC
P42	pNcGRA7_insert Gibson REV	TTTAGGAGGACACTGAGACG
P43	Tir1_vector Gibson for marker FWD	CGAGAAGTAATTAATCACCGTTGTGCTC
P44	Tir1_vector Gibson for marker REV	GTTTGGACGCATGCATACTCGTCAAAAAAC
P45	TgHXGPRT_insert Gibson FWD	GAGTATGCATGCGTCCAAACCCATTGAAG
P46	TgHXGPRT_insert Gibson REV	CGGTGATTAATTACTTCTCGAACTTTTTGCG
P47	NCLIV_056045 5' UTR check FWD	GATACTGCAGCTACGAAGCG
P48	pNcGRA7-TgHXGPRT check REV	GAACTGCTTGCGAGACCTG
P49	NCLIV_056045 coding 1st exon check REV	GGATTGTTCGTTCTGCAGCTT
P50	NCLIV_056045 coding 3rd exon check FWD	TCGACTAAGTGTCGCGTGAAT
P51	NCLIV_056045 coding 3rd exon check REV	CGTGTTCGGCGCATTTCATC
P52	NCLIV_056045 3' UTR check REV	GAGACTGCATGTCGGTTAGAG
P53	NCLIV_069360 5' UTR check FWD	GAGTGATGGCCAAACGACG
P54	NCLIV_069360 coding check FWD	GAAGCCAAGCGCAGCATTG
P55	NCLIV_069360 coding check REV	CTGCCGCATATGTTGCTGG
P56	NCLIV_066730 5' UTR check FWD	CCTCAAAGTTCGTGCGTCG
P57	NCLIV_066730 coding check FWD	CCCACGCAGAAAGTCAAGG
P58	NCLIV_066730 coding check REV	GGGAATTGGGACTGTTGGC
